# DM-PhyClus: a Bayesian phylogenetic algorithm for infectious disease transmission cluster inference

**DOI:** 10.1186/s12859-018-2347-3

**Published:** 2018-09-14

**Authors:** Luc Villandré, Aurélie Labbe, Bluma Brenner, Michel Roger, David A Stephens

**Affiliations:** 1 Department of Epidemiology, Biostatistics, and Occupational Health, McGill University, 1020 avenue des Pins Ouest, Montreal, H3A 1A2 QC Canada; 2 Department of Decision Science, HEC Montréal, 3000, chemin de la Côte-Sainte-Catherine, Montreal, H3T 2A7 QC Canada; 3 McGill AIDS Centre, Lady Davis Institute, Jewish General Hospital, 3755 chemin de la Côte-Sainte-Catherine, Montreal, H3T 1E2 QC Canada; 4 Centre de Recherche du Centre Hospitalier de l’Université de Montréal (CRCHUM), 900 rue Saint-Denis, Pavillon R, Montreal, H2X 0A9 QC Canada; 5 Département de microbiologie, infectiologie et immunologie, Université de Montréal, 2900 boul. Edouard-Montpetit, Montreal, H3T 1J4 QC Canada; 6 Department of Mathematics and Statistics, McGill University, 805 rue Sherbrooke Ouest, Montreal, H3A 0B9 QC Canada

**Keywords:** Phylogenetics, Clustering, HIV-1, Bayesian inference, Markov Chain Monte Carlo

## Abstract

**Background:**

Conventional phylogenetic clustering approaches rely on arbitrary cutpoints applied a posteriori to phylogenetic estimates. Although in practice, Bayesian and bootstrap-based clustering tend to lead to similar estimates, they often produce conflicting measures of confidence in clusters. The current study proposes a new Bayesian phylogenetic clustering algorithm, which we refer to as *DM-PhyClus* (*Dirichlet-Multinomial Phylogenetic Clustering*), that identifies sets of sequences resulting from quick transmission chains, thus yielding easily-interpretable clusters, without using any ad hoc distance or confidence requirement.

**Results:**

Simulations reveal that DM-PhyClus can outperform conventional clustering methods, as well as the Gap procedure, a pure distance-based algorithm, in terms of mean cluster recovery. We apply DM-PhyClus to a sample of real HIV-1 sequences, producing a set of clusters whose inference is in line with the conclusions of a previous thorough analysis.

**Conclusions:**

DM-PhyClus, by eliminating the need for cutpoints and producing sensible inference for cluster configurations, can facilitate transmission cluster detection. Future efforts to reduce incidence of infectious diseases, like HIV-1, will need reliable estimates of transmission clusters. It follows that algorithms like DM-PhyClus could serve to better inform public health strategies.

**Electronic supplementary material:**

The online version of this article (10.1186/s12859-018-2347-3) contains supplementary material, which is available to authorized users.

## Background

The collection and, often public, availability of viral genotyping data has made phylogenetics, the field concerned with the inference from genetic data of the ancestral history of organisms, a popular tool for modelling epidemics [1, 2]. Phylogenetic models represent the ancestral relationships between sequences of nucleotides or amino acids with a hierarchical tree structure known as a *phylogeny*. Phylogenetics can help guide public health efforts to curb incidence of HIV-1 and tuberculosis [3–5], by revealing the existence of *transmission clusters*, epidemiologically-linked individuals infected by a genetically-similar pathogen. Transmission clusters are known to affect incidence and may hinder the implementation of effective intervention strategies [6].

### Transmission cluster inference

Observed clustering in viral sequencing data, thought to result from series of fast onward transmission events called *quick transmission chains*, is a convenient proxy for transmission clusters [7]. To estimate transmission clusters from an inferred phylogeny, a collection of ad hoc rules are conventionally applied. One normally looks for a partition of the sample into *clades*. A clade is a set of sequences corresponding to all tips descended from a given ancestral node in the tree. Usually, a clade corresponds to a cluster only when it is known with high confidence, and when its sequences are similar. Unsurprisingly, disagreements over clustering rules are common, and what the resulting partitions mean in an epidemiological sense is still unclear [8, 9].

### Study objective

In the present study, we aim to propose a new Bayesian phylogenetic clustering algorithm, called *DM-PhyClus*, which stands for *Dirichlet-Multinomial Phylogenetic Clustering*, that eliminates the need for arbitrary distance and confidence criteria. DM-PhyClus looks directly for sets of sequences resulting from quick transmission chains, thus also improving interpretability of clusters.

### Phylogenetic inference and clustering

Bayesian phylogenetic inference is commonly used in the clustering of sequencing data, mainly because it readily provides an intuitive confidence measure for inferred clades [10, 11]. Popular software implementations include BEAST and MrBayes [11, 12], which both rely on variations of the Markov Chain Monte Carlo (MCMC) approach. Convergence issues have prompted the development of several other approaches, based, for example, on Sequential Monte Carlo [13] and Stochastic Approximation Monte Carlo [14].

Software like MEGA and PAUP* [15, 16] have made maximum likelihood (ML) phylogenetic reconstruction a popular alternative. RAxML [17] and FastTree [18] are more recent options, designed specifically to handle large datasets. They both rely on heuristic tree-searching strategies to considerably speed up likelihood optimization. Generally, methods for maximum likelihood phylogenetic reconstruction do not yield measures of confidence for clades, which are necessary to apply conventional clustering rules. To solve that problem, they are combined with a bootstrap scheme. However, the interpretation of bootstrap support for clades remains controversial [19–21].

Bayesian and ML phylogenetic approaches involve generating a large collection of trees. The maximum posterior probability (MAP) or ML estimate are natural choices for the tree that best describes the ancestry of the data. However, especially in large samples, the score for those estimates may not be much higher than that for many other trees. Therefore, summarizing a collection of phylogenies by building a so-called *consensus tree* [22–24] is common. Unlike conventional point estimates, consensus trees provide measures of uncertainty for elements in the tree *topology*, an unambiguous representation of the hierarchical nesting of clades in the phylogeny.

After computing a sensible phylogenetic estimate, one can then proceed to estimate clusters. [7] define a cluster as a clade known with high confidence, and with *patristic distances* bounded above by a reasonably low value, where the patristic distance between any two sequences is calculated by summing branch lengths along the path linking the corresponding tips in the tree. The method itself however does not specify how confidence and distance requirements should be selected. In their ML-bootstrap analysis for example, [7] used a confidence threshold of 98% and a patristic distance requirement of 0.015 nt/bp.

Prosperi et al. [25] designed PhyloPart, a method that also defines clusters as clades known with high confidence. The genetic distance requirement is now formulated in terms of the median patristic distance in a clade. To conclude in clustering, we must have median patristic distance in a clade below a value equal to a reasonably low percentile of patristic distances in the entire tree. In their analyses, [25] used the 1st, 10th, 15th, and 30th percentiles. The choice of a percentile threshold is arbitrary: in their study, it was selected to maximize agreement with a number of confirmed clusters.

Alternatively, [26] proposed ClusterPicker, that also finds clusters by identifying clades inferred with reasonably high confidence. The distance requirement in ClusterPicker does not involve patristic distances, but rather simple pairwise estimates of genetic distance, computed for example with the JC69, K80, HKY85, or raw (Hamming) model [27–29]. The method is convenient, as it can be applied readily to consensus trees, which do not naturally have branch lengths. Once again, the tuning of the clustering requirements is left entirely to the investigator.

Clustering criteria are often arbitrary, and tend to be poorly justified. In Bayesian phylogenetic clustering, posterior probability requirements of 1 are the most common [19, 30], although studies may opt for a lower value [31]. In the ML-bootstrap framework, clade support requirements as low as 70% [32–34], or above 90% [7, 26, 35] are common. A lot of variability is also observed in genetic distance requirements. For instance, [35] use the HKY+ *γ* model [29] to assess pairwise distances between sequences and impose a maximum distance of 0.045 nt/bp within any cluster. [25] instead find that a median patristic distance requirement of 0.07 nt/bp maximizes correspondence with known clusters.

The variety of standards encountered in the literature may reflect a lack of agreement as to what clusters correspond to [8]. More recently, [36] proposed the *Gap Procedure*, a distance-based clustering approach that avoids phylogenetic reconstruction and cutpoint selection altogether by defining clusters based on a measure of distinctiveness. Although it is very fast, it does not provide any means to evaluate uncertainty around its point estimates. Like the Gap Procedure, the method presented in this paper aims to avoid cutpoint selection by giving clusters a straightforward definition. However, it should also offer an intuitive measure of confidence in cluster estimates. We designed it specifically for clustering HIV-1 sequencing data, which will be the main substantive focus in the remainder of the paper.

## Methods

**Fig. 1:**
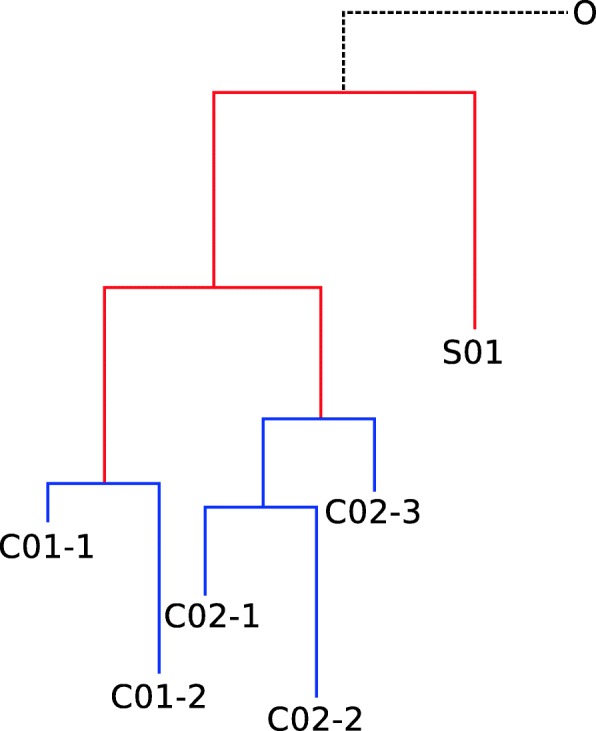
A phylogeny split into between- and within- cluster components. Sequences C01-1 and C01-2 belong to cluster 1, while C02-1, C02-2, and C02-3 belong to cluster 2. Sequence S01 is a singleton, that is, a cluster of size 1, and O is an outgroup, used to root the sample phylogeny. The red sub-phylogeny is called the *between-cluster* phylogeny, while the blue sub-phylogenies are called the *within-cluster* phylogenies

DM-PhyClus is a MCMC-based algorithm [37] that innovates by relying on a definition of transmission clusters that better reflects clinical understanding, and by avoiding ad hoc distance and confidence requirements. DM-PhyClus makes use of a likelihood formulation that distinguishes between *between-cluster* and *within-cluster* components of the phylogeny, cf. Fig. 1. The between-cluster phylogeny represents the ancestral relationships between each cluster’s most recent common ancestor (MRCA), and the within-cluster phylogenies, the ancestral history of each cluster.

Under DM-PhyClus, clusters have a clear definition: they are sets of sequences whose ancestral history is characterized by a specific distribution for branch lengths. In order for clusters to reflect quick transmission chains, we attribute branch lengths in the within-cluster phylogenies a prior with a reasonably low mean, in comparison to that for branches in the between-cluster phylogeny.

### Likelihood

We compute the tree likelihood recursively with Felsenstein’s tree-pruning algorithm [38]. Let (*y*_1_,…,*y*_*n*_) denote the sequence data, and *y*_*i*,*s*_, the state at the *s*’th site, *s*=1,…,*S*, of sequence *i*. If sequences are made up of nucleotides, *y*_*i*,*s*_ can take one of 4 values, each represented by a unit vector of length 4. For example, nucleotides *A* and *T* are represented by vectors (1,0,0,0) and (0,1,0,0), respectively.

At each site, evolution along branches of the tree, whose topology and branch lengths are denoted *τ* and *l*, respectively, follows a continuous time Markov chain with rate matrix *Q*. Further, we assume that among-sites variation in evolution rates follows a discrete gamma distribution with *n*_*r*_ categories and parameter *r*. Evolution occurs independently at different sites and so, the likelihood takes value,

1 $$ \zeta\left(\tau, \mathbf{l}, n_{r}, r, Q\right) = \prod\limits_{s = 1}^{S} \zeta_{s}\left(\tau, \mathbf{l}, n_{r}, r, Q\right),  $$

where *ζ*_*s*_(*τ*,**l**,*n*_*r*_,*r*,*Q*) represents the likelihood contribution of site *s*.

Let *j* and *k* index the two children of an arbitrary internal node *i* in topology *τ*, and *x*_._ be a numerical code for the state at node., e.g. *A*=1, *T*=2, *C*=3, *G*=4. Let $L_{x_{i}}^{(s,i,m)}$ represent the probability of observing the configuration at all tips descended from parent node *i* at site *s*, conditional on state *x*_*i*_ at that node and rate variation category *m*. We have that,

2 $$ {{}\begin{aligned} L_{x_{i}}^{(s,i,m)} =\! \sum_{x_{j}} p_{x_{i},x_{j}}\! \left(\xi_{m} l_{j}\right)L_{x_{j}}^{(s,j,m)}\! \sum_{x_{k}} p_{x_{i}, x_{k}}\left(\xi_{m} l_{k}\right)L_{x_{k}}^{(s,k,m)}, \end{aligned}}  $$

where $p_{x_{i},x_.}\left (\xi _{m} l_.\right)$ represents the transition probability from state *x*_*i*_ to *x*_._ along a branch of length *l*_._, with coefficient *ξ*_*m*_ being a scaling factor resulting from the conditioning on rate variation category *m*. We note that *x*_*i*_ indexes the **L**^(*s*,*i*,*m*)^ vector, and it follows that the vector has as many elements as there are states in the data, e.g. 4 for nucleotide data. From the Markov assumption, it follows that,

$$p_{x_{i},x_.}\left(\xi_{m} l_.\right) = \exp\left(Q\xi_{m} l_.\right). $$

When index *i* is for a tip, we have that **L**^(*s*,*i*,*m*)^=*y*_*i*,*s*_. We must compute **L**^(*s*,*i*,*m*)^ for each combination of site *s*, node *i*, and rate variation category *m*.

We start by computing **L**^(*s*,*i*,*m*)^ for all nodes *i* whose children *j* and *k* are both tips. Then, we list all pairs of nodes *j* and *k* for which both **L**^(*s*,*j*,*m*)^ and **L**^(*s*,*k*,*m*)^ are known, and compute **L**^(*s*,*i*,*m*)^ for each of them.

Let the root of the tree have index *𝜗*. We have that the likelihood contribution of site *s* takes value,

$$\zeta_{s}(\tau, \mathbf{l}, n_{r}, r, Q) = \frac{1}{n_{r}}\sum\limits_{m = 1}^{n_{r}} \sum_{x_{\vartheta}} L_{x_{\vartheta}}^{(s,\vartheta,m)}p_{x_{\vartheta}}, $$

where **p** represents the limiting probabilities of the Markov chain.

In real DNA sequences, sequencing may reveal that two or more nucleotides can be found at certain sites, producing an *ambiguity*. In the case of HIV, the sequences used when fitting phylogenetic models are summaries of the viral population found within infected individuals. Ambiguities are especially common in such sequences, since genetic diversity in the within-host viral population increases quickly after the infection event took place [39]. In Felsenstein’s tree-pruning algorithm, ambiguities are expressed as a sum of the unit vectors for the potential states. For example, if A and T are observed at site *m* in sequence *i*, we get that *y*_*i*,*m*_=[1,1,0,0].

### Priors

We denote branch lengths in the within-cluster and between-cluster components **l**^(*w*)^ and **l**^(*b*)^, respectively. We assign branch lengths in the between-cluster phylogeny a log-normal prior with parameters *μ* and *σ*. We picked that distribution because of its potentially heavy right tail, which allows for a small number of distinctively long branches. We tune priors for those parameters based on a desired mean and coefficient of variation. To lighten the computational load, we assign that mean a uniform prior over a finite number of discrete values, and the coefficient of variation is fixed. We assign branch lengths in within-cluster phylogenies an exponential prior with rate *δ*, whose prior is, like before, discrete uniform over a finite range of sensible values.

We assign cluster membership indices **c**=(*c*_1_,…,*c*_*n*_) a multinomial prior with probability parameters ***π***=(*π*_1_,…,*π*_max(**c**)_), weighted by values from a Poisson distribution, with rate parameter *λ*, evaluated at max(**c**) and an indicator function giving probability 0 to configurations not meeting the clade assumption,

3 $$ {{\begin{aligned} P(c_{1}, \dots, c_{n} \mid \tau, \lambda, \boldsymbol{\pi}) &\propto \binom{n}{n_{1} \dots n_{\max(\mathbf{c})}} \pi_{1}^{n_{1}} \cdots \pi_{\max(\mathbf{c})}^{n_{\max(\mathbf{c})}} \frac{\exp(-\lambda)\lambda^{\max(\mathbf{c})}}{\max(\mathbf{c})!} \times \\ & \times I[\text{Partition allowed by \(\tau\)}], \end{aligned}}}  $$

with $n_{k} = {\sum \nolimits }_{i=1}^{n} I[c_{i} = k]$ and *I*[.] being an indicator function.

**Fig. 2:**
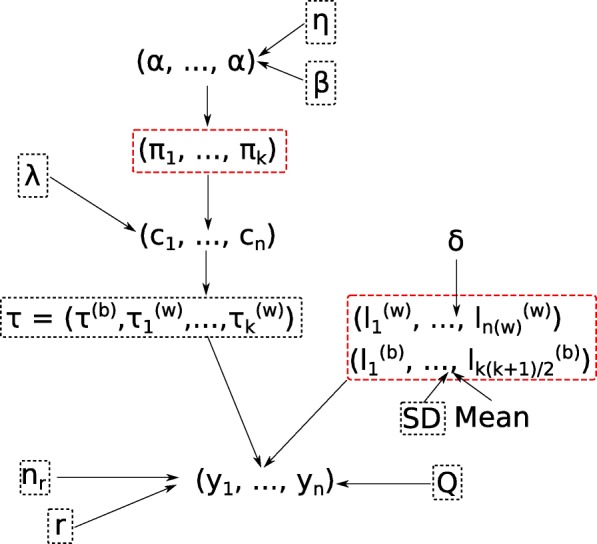
Graphical representation of the relationships between parameters and the data. Parameters in a black box are fixed. Parameters in a red box are marginalized out. The vector (*y*_1_,…,*y*_*n*_) is the sample, and “SD” stands for standard deviation. We denote the within-cluster phylogenies $\left (\tau ^{(w)}_{1}, \dots, \tau ^{(w)}_{k}\right)$, *k* being the number of clusters, and the between-cluster phylogeny, *τ*^(*b*)^. Within-cluster phylogenies are degenerate when they support a cluster of size 1, while the between-cluster phylogeny is degenerate when the sample comprises only one cluster. The log-normal prior distribution for the between-cluster branch lengths is reparameterized in such a way that it has mean and standard deviation parameters, like in the normal distribution

The probability parameters have a symmetric Dirichlet hyperprior with concentration parameter *α*, to which we assign a gamma hyperprior with shape and scale parameters *η* and *β*. We summarize parameters in Fig. 2 and present more information on the algorithm’s input, e.g. parameters, priors, hyperpriors and tuning parameters, and outputs in Appendix 1.

### Posterior probability derivation

We are interested primarily in the posterior distribution of cluster membership indices **c** and so, we marginalize out probability parameters ***π***, as well as all branch lengths. Marginalizing out ***π*** from Eq. 3, we obtain,

4 $$ {{\begin{aligned} P(c_{1}, \dots, c_{n} \mid \tau, \boldsymbol{\alpha}, \lambda) &\propto \frac{B(\mathbf{n} + \boldsymbol{\alpha})}{B(\boldsymbol{\alpha})}\binom{n}{n_{1} \dots n_{\max(\mathbf{c})}} \frac{\lambda^{\max(\mathbf{c})} \exp(-\lambda)}{\max(\mathbf{c})!} \times \\ &\times I[\text{Partition allowed by \(\tau\)}],  \end{aligned}}}  $$

with,

$$B(\boldsymbol{\alpha}) = \frac{\prod_{i=1}^{\max(\mathbf{c})} \Gamma(\alpha_{i})}{\Gamma\left(\sum_{i=1}^{\max(\mathbf{c})} \alpha_{i}\right)}. $$

We use Monte Carlo integration to marginalize out branch lengths from the likelihood. When the number of Monte Carlo replications *K* is large enough, the probability of a transition from state *x*_*i*_ to *x*_*j*_ over any given branch is approximately,

5 $$ {\begin{aligned} P(x_{j} \mid x_{i}, \mathbf{c}) = \int_{\mathcal{D}(l\mid\mathbf{c})}\!\! \left[\exp(Ql)\right]_{(x_{i},x_{j})} p(l \mid \mathbf{c}) dl \approx \frac{1}{K}\sum_{k = 1}^{K} \left[\exp(Ql_{k}) \right]_{(x_{i},x_{j})},  \end{aligned}}  $$

where $\mathcal {D}(l\mid \mathbf {c})$ is the domain of *l*∣**c**, *p*(*l*∣**c**) is the prior distribution of *l* conditional on **c**, and *l*_*k*_ is drawn from that distribution. [ exp(*Ql*)] denotes the transition probability matrix along a branch of length *l*, and $[\exp (Ql)]_{(x_{i},x_{j})}$ represents element (*x*_*i*_,*x*_*j*_) of that matrix. The conditioning on **c** appears as a result of the marginalization, because of the different priors for branch lengths in the within-cluster phylogenies and the between-cluster phylogeny.

The posterior distribution of the cluster membership indices is denoted,

$${\begin{aligned} P(c_{1}, \ldots, c_{n} \!\mid\! y_{1}, \ldots, y_{n}, \tau, \boldsymbol{\alpha}, \lambda) \propto \zeta(\tau, n_{r}, r, Q \!\mid\! c_{1}, \ldots, c_{n}) P(c_{1}, \ldots, c_{n} \!\mid\! \tau, \boldsymbol{\alpha}, \lambda), \end{aligned}} $$

where *P*(*c*_1_,…,*c*_*n*_∣*τ*,***α***,*λ*) is given by Eq. 4 and *ζ*(*τ*,*n*_*r*_,*r*,*Q*∣*c*_1_,…,*c*_*n*_) is obtained by replacing $p_{x_{i},x_.}(\xi _{m} l_.)$ in Eq. 2 by the approximation derived in Eq. 5, but with simulated branch lengths *l*_*k*_ being multiplied by *ξ*_*m*_. There is a one-to-one correspondence between (*c*_1_,…,*c*_*n*_) and the breakdown of *τ* into within-cluster phylogenies and between-cluster phylogeny, and the conditioning on (*c*_1_,…,*c*_*n*_) in the marginal likelihood appears as a result.

### Transition kernels and Metropolis-Hastings (MH) ratios

DM-PhyClus first searches for a sensible phylogenetic estimate, that acts to restrict the space of potential cluster membership indices, and then, conditional on that phylogeny, performs successive Metropolis-Hastings (MH) updates of the concentration parameter and the cluster membership indices.

We sample tentative transitions in the space of concentration parameter *α* from a uniform distribution defined over an interval of length 1 centered around the current value of *α*, resulting in the transition kernel ratio reducing to 1. We propose moves in the space of cluster membership indices **c** by using a cluster split-merge strategy. Any cluster of size 2 or more can be split in two disjoint clusters, corresponding to the clades supported by the children of the original cluster’s root. We can merge any two neighbouring clusters, or in other words, any two clusters whose most recent common ancestor is at most one split above their respective roots. The transition kernel is a discrete uniform distribution over all split-merge transitions allowed by the topology from the current state. It follows that the transition kernel ratio is equal to the total number of potential moves from the current configuration divided by the total number of potential moves starting from the proposal. With the ratio of priors obtained from Eq. 4 and the conventional likelihood ratio, we have all necessary components for computing the MH ratio.

### Point estimates for cluster membership indices

We produce two kinds of estimates for cluster membership indices, the *maximum posterior probability* (MAP) estimate, and the *linkage-xx* estimate, which we obtain in three steps,

1. Derive an *adjacency matrix* from each sampled cluster membership indices vector.
  An adjacency matrix is a symmetrical matrix with a 1 at position (*i*,*j*) if elements *i* and *j* co-cluster, and with a 0 otherwise.
2. Average adjacency matrices computed in step 1 and apply a co-clustering frequency threshold of *xx*.
  The average adjacency matrix provides co-clustering frequencies. All frequencies higher than the threshold are rounded up to 1, while all others are rounded down to 0.
3. Identify all *disjoint* sets, called *modules* or *components*, from the matrix obtained in step 2.
  Two sets of sequences are disjoint if no co-clustering exists between them. We use the walktrap algorithm [40] to detect disjoint sets, which leads to the cluster estimates.

We present a structured, step-by-step description of DM-PhyClus in Appendix 1.

### Simulation study

#### Data

We simulate an HIV-1 sequence dataset of size 200 by going through the following steps:

1. Sample the total number of clusters from a Poisson distribution with mean 50,
2. Sample cluster assignment probabilities from a symmetric Dirichlet distribution with a concentration parameter generated from a normal distribution with mean 10 and standard deviation 2,
3. Sample 200 values from a multinomial distribution with the obtained probability vector,
4. Generate each within-cluster phylogeny by picking a topology at random, and by sampling branch lengths from an exponential distribution with mean equal to 0.003,
5. Generate the between-cluster phylogeny by picking a topology at random, and by sampling branch lengths from a log-normal distribution with mean and standard deviation equal to 0.008,
6. Let the *HXB2* sequence evolve along the simulated tree, with evolution rate matrix and limiting probabilities obtained from [41].

HXB2 is an HIV-1 subtype B sequence that serves as a reference for site position numbers in any HIV-1 sequence. In other words, the range of site indices in any HIV-1 sequence is found by aligning it with HXB2. We generate 50 datasets in total, and add to each of them an arbitrary subtype C outgroup (http://www.hiv.lanl.gov/, accession number: AB254141) for rooting the inferred phylogenies. We list parameters used for data generation in Appendix 2.

#### Scenarios

Assessing sensitivity of the cluster estimates to the concentration parameter prior is vital, as it may be challenging to properly specify in practice. For each simulated dataset, we run DM-PhyClus under the assumption that the concentration parameter follows a gamma distribution with scale parameter 0.1, and, successively, with means 1, 10, and 100. The use of fixed estimates for the mutation rate matrix and limiting probabilities may also affect cluster recovery. To verify that such a restriction is not overly detrimental to cluster recovery, we use values for those parameters obtained from a separate analysis of a real HIV-1 sequence dataset, that we ensure are reasonably different from those used for data generation.

#### Setup

Given the synthetic nature of the problem, tuning priors for branch lengths is difficult and so, we opt for an empirical Bayes approach. We setup a first round of simulations with the following scheme:

**For each simulated dataset**:

1. Use RAxML [17] to derive a maximum likelihood phylogenetic estimate and perform 500 bootstrap iterations, producing the usual clade support estimates,
2. Obtain an initial set of clusters by running a depth-first search on the ML tree: stop exploration along any path once you find a clade with bootstrap support greater than 70% and with patristic distances below a certain threshold, selected through maximization of the Dunn index [42], a measure of clustering quality,
3. Derive mean branch lengths for the within- and between-cluster phylogenies,
4. Define a range around each of the two means obtained previously with radius equal to 8% of the obtained value,
5. Select 20 equidistant points in each range, at which transition probability matrices are computed by sampling 100,000 values from the log-normal distribution for between-cluster branch lengths, or the exponential distribution for within-cluster branch lengths,
6. Use the initial set of clusters as a starting value for the chain in DM-PhyClus, and use the maximum likelihood topology to bound the space of cluster membership indices.

We made the decision to use only 20 points to increase computational speed. In light of that choice, we then selected the 8% radius to ensure that transitions in the space of transition probability matrices were likely enough.

In a second round of simulations, before launching the chain, we explore the topological space around the maximum likelihood phylogeny, using nearest-neighbour interchange to find a configuration that improves posterior probability, and letting values for the concentration parameter and cluster membership indices vary as well. We start the MCMC run once a suitable topology is identified. We present an exhaustive list of the tuning parameter values used in the simulations in Appendix 2.

#### Chain configuration and point estimates comparison

For each simulated dataset, we produce 55,000 samples from the posterior distribution of the cluster membership indices vector. We apply a thinning ratio of 1 over 50, and take out the first 5000 iterations as a burn in, leaving us with 1000 samples. Once the MCMC run is complete, we obtain the MAP and linkage-*xx* cluster estimates, and measure overlap between the real and inferred clusters with the adjusted Rand Index (ARI), a measure of similarity between two sets of clusters. It involves the ratio of pairs of elements that are similarly co-clustered or dissociated in both sets to the total number of pairs in the sample, combined with a numerical adjustment for chance. It is bounded above by 1, which indicates perfect correspondence. We compare those estimates to those we initially obtained from RAxML, which we refer to as the *Bootstrap-70* estimates, and to the estimates from the so-called *Gap procedure*, a quick distance-based genetic sequence clustering approach that requires minimal tuning [36]. The Bootstrap-70 estimate is a natural standard for comparison, since it is obtained by applying a conventional method for the clustering of HIV-1 sequencing data [19].

### Real data analysis

#### Data

The original sample consists of 3537 HIV-1 subtype B sequences collected for the Québec HIV genotyping program [7]. Each sequence is from a different male patient belonging to the injection drug user (IDU) or men who have sex with men (MSM) risk category, and that has not yet started antiretroviral therapy, the standard treatment regimen for HIV-positive individuals. The dataset includes sites 10–297 of the protease region (PR), and 112–741 of the reverse transcriptase (RT) region, of the *pol* gene.

Brenner et al. [6] obtained an initial set of clusters by partitioning the sample through inspection of the maximum likelihood tree, selecting clades with bootstrap support greater than 98% and whose patristic distances were below 0.01 nt/bp. They also looked for congruent polymorphisms and mutational motifs. Whenever new sequences entered the database, they updated their cluster estimates by re-inferring the tree, and attaching new sequences to previously-inferred clusters when the clade they belonged to had bootstrap support greater than 98%. They also used clinical and demographic information to exclude sequences from inferred clusters.

We focus on a subsample of 526 sequences, made up of 18 previously-inferred clusters of sizes ranging from 2 to 69, inclusively, as well as 12 singletons selected uniformly at random in the original sample. We add to the sample 3 subtype C outgroups from Zambia, downloaded from the Los Alamos HIV-1 sequencing database (http://www.hiv.lanl.gov/, accession numbers AB254141, AB254142, AB254143).

#### Bootstrap analysis

To evaluate sensitivity of DM-PhyClus to the input topology, we produce 100 bootstrap samples of the data by resampling site indices with replacement and re-assembling each sequence based on the sampled indices. We use maximum likelihood topological estimates and use the same strategy as in the simulations to obtain starting values for the chain. Each run also consists of 55,000 iterations, with a burn-in of 5000 and a thinning ratio of 1/50.

#### Approximation of the fully Bayesian analysis

Fixing the topological parameter in the chain results in the inference not being fully Bayesian. Such an approximation is acceptable only so long as we can establish that the results do not differ too much from those resulting from the fully Bayesian approach. To do so, we first use MrBayes [11], run under the default configuration, to sample 1.5 million phylogenies from the posterior distribution *P*(*τ*∣**y**,…), where … represents the other parameters. We take out the first 375,000 phylogenies as a burn-in, and apply a thinning ratio of 1/500. Of the remaining 2250 phylogenies, we select 100 uniformly at random, which we use as input in 100 separate runs of DM-PhyClus. Each run produces samples from the conditional posterior distribution of the cluster membership indices *P*(**c**∣*τ*_*i*_,…,**y**), *i*=1,…,100. Noting that,

$$ P(\mathbf{c} \mid \mathbf{y}) = E_{\tau}[P(\mathbf{c} \mid \tau, \mathbf{y})] \approx \sum_{i = 1}^{100} P(\mathbf{c} \mid \tau_{i}, \mathbf{y})/100, $$

we see that high overlap between the maximum posterior probability cluster membership indices obtained from the 100 chains ensures that the peak of *P*(**c**∣**y**) is found at a configuration similar to those obtained in each individual run, thus confirming the quality of the approximation resulting from the conditioning assumption.

#### Main run

We obtain starting values with the help of RAxML, under the assumption that genetic distance follows the GTR + *Γ*(3) model. As in the simulations, we configure priors for branch lengths based on the maximum likelihood topology. We use limiting probabilities and nucleotide substitution rates previously inferred for HIV-1 subtype B [41]. We assume discrete gamma substitution rate variation with 3 categories. Finally, we fix the rate parameter for the Poisson distribution at 30, the number of clusters obtained in [6]. We run 220,000 iterations, keeping one iteration out of 150 and taking out the first 70,000 iterations as a burn-in. We then obtain point estimates for cluster membership indices as before. An exhaustive list of tuning parameter values used in all real data analyses is available in Appendix 3. With this analysis, we aim to highlight the extent to which the cluster estimates produced by DM-PhyClus differ from those presented in [6], that were based on a conventional analysis relying on ad hoc cutpoints. Our choice of Poisson and evolutionary parameters aims to reflect our prior understanding of the clustering in the data as best as possible.

### Software

We present a technical description of the software in Appendix 4. We implement the algorithm in R, with functions contained in the *phangorn*, *ape*, and *phytools* libraries [43, 44]. Likelihood evaluations rely on compiled C++ code integrated into the R script using the *Rcpp* and *RcppArmadillo* packages [45, 46]. We produce starting values with RAxML [17]. Finally, we also produce cluster estimates with the the GapProcedure package [36]. A package, *DMphyClus*, is available on Github (https://github.com/villandre/DMphyClus) and will be submitted to CRAN.

## Results

### Simulation study

On an Intel(R) Xeon(R) CPU E7-4820 v4 2.00GHz CPU, running 55,000 iterations took on average a bit more than 2 hours. Log-posterior probability graphs show no obvious issue with autocorrelation or convergence, and indicate good mixing (see, for example, Appendix 5). We show the obtained ARIs for the six scenarios in Table 1 (the raw data used to produce the tables are available as Additional files 1, 2, 3, 4, 5 and 6). Overall, mean cluster recovery from DM-PhyClus was superior than that from the conventional Bootstrap-70 approach and GapProcedure, both of which usually struggled to recover the clusters. We observe a noticeable drop in mean overlap when the concentration parameter has a prior whose mean is much smaller than that used for data generation, but not when it is larger.

**Table 1:** Summary statistics for adjusted Rand indices (ARI) for cluster membership estimates obtained from chains run on 50 datasets under different simulation scenarios

Topology used	Alpha mean	Estimator	Min.	Max.	10th perc.	Median	90th perc.	Mean	SD	SE
GapProcedure	-	-	0.012	0.719	0.030	0.385	0.654	0.361	0.227	0.005
Bootstrap-70	-	-	0.074	0.882	0.256	0.483	0.771	0.504	0.221	0.004
ML topology	10	MAP	0.000	0.935	0.686	0.820	0.900	0.769	0.210	0.004
		Linkage-0.7	0.000	0.946	0.711	0.853	0.920	0.793	0.213	0.004
		Linkage-0.8	0.000	0.971	0.707	0.838	0.912	0.793	0.213	0.004
		Linkage-0.9	0.000	0.962	0.710	0.822	0.893	0.771	0.206	0.004
		Linkage-1	0.089	0.710	0.359	0.494	0.631	0.484	0.129	0.003
	1	MAP	0.098	0.862	0.328	0.619	0.833	0.601	0.199	0.004
		Linkage-0.7	0.012	0.939	0.381	0.725	0.861	0.653	0.218	0.004
		Linkage-0.8	0.011	0.959	0.394	0.760	0.865	0.680	0.207	0.004
		Linkage-0.9	0.053	0.937	0.466	0.776	0.885	0.712	0.191	0.004
		Linkage-1	0.159	0.716	0.397	0.470	0.646	0.491	0.103	0.002
	100	MAP	0.123	0.931	0.594	0.848	0.917	0.790	0.196	0.004
		Linkage-0.7	0.123	0.973	0.346	0.859	0.931	0.791	0.215	0.004
		Linkage-0.8	0.123	0.971	0.348	0.852	0.920	0.785	0.211	0.004
		Linkage-0.9	0.123	0.980	0.378	0.820	0.896	0.761	0.202	0.004
		Linkage-1	0.123	0.802	0.351	0.514	0.652	0.504	0.133	0.003
MAP topology	10	MAP	0.000	0.935	0.714	0.839	0.923	0.798	0.180	0.004
		Linkage-0.7	0.000	0.950	0.727	0.858	0.919	0.818	0.172	0.004
		Linkage-0.8	0.000	0.953	0.791	0.846	0.919	0.823	0.165	0.003
		Linkage-0.9	0.000	0.947	0.751	0.824	0.891	0.798	0.156	0.003
		Linkage-1	0.000	0.686	0.318	0.449	0.598	0.454	0.117	0.002
	1	MAP	0.011	0.870	0.329	0.623	0.832	0.598	0.203	0.004
		Linkage-0.7	0.162	0.930	0.321	0.738	0.848	0.649	0.212	0.004
		Linkage-0.8	0.170	0.931	0.384	0.746	0.872	0.671	0.201	0.004
		Linkage-0.9	0.175	0.911	0.437	0.764	0.852	0.693	0.178	0.004
		Linkage-1	0.341	0.745	0.396	0.516	0.660	0.524	0.093	0.002
	100	MAP	0.123	0.947	0.761	0.854	0.914	0.816	0.171	0.003
		Linkage-0.7	0.123	0.976	0.793	0.867	0.923	0.830	0.170	0.003
		Linkage-0.8	0.123	0.970	0.789	0.857	0.914	0.825	0.169	0.003
		Linkage-0.9	0.123	0.965	0.703	0.819	0.901	0.789	0.164	0.003
		Linkage-1	0.123	0.672	0.298	0.459	0.619	0.457	0.122	0.002

The linkage-*xx* estimates performed comparably or slightly better than the MAP estimates when the linkage requirement was 0.7−0.8 and the prior on the concentration parameter had mean equal or superior to the the true value. When the prior underestimated the true concentration parameter value however, the linkage estimates greatly improved recovery, sometimes as much as 10%, as long as the linkage requirement was not 1. Maximum observed recovery rates were also consistently superior for the linkage estimates.

The slightly better performance of DM-PhyClus when the concentration parameter has a mean greater than that used for data generation was unexpected. We observe it both when the MAP and ML topologies are used. When the concentration parameter prior had mean 10, two chains returned a MAP configuration with a single cluster, producing the 0 in the table, which explains at least part of the gap. The datasets analysed by those chains seem to imply a hard clustering problem, as evidenced by the low recovery rates from Bootstrap-70, 0.13 and 0.18. Overall, starting with the MAP configuration from a shorter preliminary run resulted in small increases in mean recovery rates. When the concentration prior mean was 10, the same two chains as before resulted in a MAP configuration with only 1 cluster, yielding ARI = 0. With median recovery around 0.87 in the better scenarios, we are not overly worried about the consequences of using fixed values for the limiting probabilities and mutation rate matrices, as long as they are selected reasonably.

### Real data analysis

#### Bootstrap analysis

We measured overlap within all pairs of MAP configurations produced in the bootstrap analysis. ARIs ranged from 0.10 to 0.98, with median 0.83 and mean 0.72, indicating reasonable robustness of the chain to the assumed topology. Unsurprisingly, linkage estimates led to essentially the same conclusion. For example, overlap between cluster configurations proposed under the linkage-0.7 estimate ranged from 0.11 to 0.98, with median 0.83 and mean 0.70. Moreover, concordance between MAP estimates from the bootstrap replicas and the MAP cluster configuration obtained from the full data was generally high, with median and mean ARI equal to 0.88 and 0.80, respectively.

#### Approximation of the fully Bayesian analysis

Estimates based on the 100 topologies sampled with MrBayes were overall very similar, leading to the conclusion that the DM-PhyClus estimates are reasonable approximations of those resulting from a fully Bayesian analysis. Indeed, concordance between the MAP estimates obtained from the 100 chains tended to be high: ARIs ranged from 0.38 to 1, with median and mean 0.89 and 0.86, respectively. Overlap with the usual MAP estimate, obtained conditional on the topology found to optimize joint posterior probability after a short exploration of the topological space, was also considerable, with median and mean 0.92 and 0.90, respectively.

#### Full data analysis

**Fig. 3:**
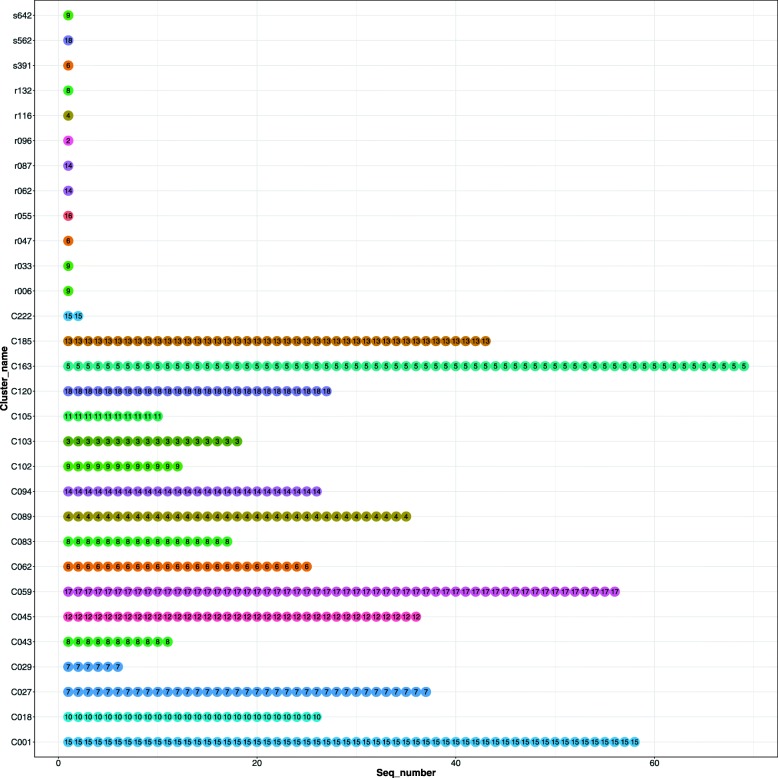
Comparison of the DM-PhyClus cluster estimates with a proposed cluster configuration for the real dataset. The coordinates on the vertical axis indicate cluster membership according to [6], and the colour and number of each dot, the cluster membership according to the maximum posterior probability (MAP) estimate of DM-PhyClus

The MAP configuration obtained from DM-PhyClus revealed the existence of 16 clusters of size 2 or more, and 2 singletons. Linkage estimates were identical to the MAP estimate when the linkage requirement was 98% or below, indicating little uncertainty in the returned partition. The Gap Procedure returned a rather similar set of clusters (ARI = 0.87). We represent clusters from DM-PhyClus against those from the curated analysis in Fig. 3. DM-PhyClus has a tendency to merge neighbouring clusters, as evidenced by the smaller number of singletons and the merger of clusters 43 and 83, which also absorbed sequence r132, and of clusters 27 and 49. The GapProcedure, on the other hand, proposed a configuration with 43 clusters of size 2 or more, and 14 singletons, splitting, for example, clusters 18 and 59 in 3 and 8 sets, respectively.

## Discussion

In this paper, we introduced a phylogenetic clustering algorithm, DM-PhyClus, that integrates an original cluster definition into cluster inference, which results in more intuitive estimates, unlike conventional approaches, that rely instead on arbitrary cutpoints applied a posteriori to a phylogenetic estimate. Simulations indicate that the algorithm can accurately recover phylogenetic clusters, often outperforming more conventional approaches. Analysis of a real dataset of HIV-1 subtype B sequences revealed a set of clusters largely similar to that from a previous analysis, but with more straightforward inference.

The study does have some limitations. Because of time constraints, we were only able to run short chains in the simulations. Log-posterior probability graphs for the simulated samples however did strongly suggest that the chains had converged, making us confident that increasing the number of iterations would not change our conclusions. We suspect that the apparent weakness of Bootstrap-70 might be in part attributable to the use of the Dunn index. For several simulated datasets, we noticed that it failed to identify the optimal partition in terms of recovery. Comparing our results to that optimal partition would have been unfair, however, since identifying it requires knowledge of the true clusters.

For computational reasons and to ensure adequate mixing in the chain, we opted for a fixed topology, thus limiting the number of partitions the algorithm can propose and ignoring uncertainty in phylogenetic reconstruction. This restricts the support of the domain for cluster membership indices, and may, as a result, inflate the posterior probability estimate for certain clusterings. Although simulations and the real data analyses indicate that this simplification works well in practice, proposing an efficient transition kernel that jointly updates cluster membership indices and the phylogeny would be necessary.

Moreover, we used RAxML mainly because of its speed. It produces a heuristic estimate of the maximum likelihood phylogeny, and it is possible that a different algorithm, such as the one in MEGA [15], might suggest a different tree. RAxML has a long development history and has been thoroughly tested, and so, we trust the quality of its estimates. It follows that we do not expect them to differ widely from those produced by comparable software.

Further DM-PhyClus rests on the assumption that cluster-specific phylogenies have a distinctive branch length distribution. Our goal was to reflect intuitive understanding of transmission clusters, but our branch length assumptions do remain simplistic. Phylogenies for HIV-1, for instance, are characterized by long external branches [39]. The presence of a few very long external branches is problematic for certain clustering algorithms that rely on a maximum distance requirement, and could potentially affect the results of DM-PhyClus. Moreover the exponential prior is known for producing overly long trees [47]. The assumption however is common in Bayesian phylogenetic inference [12], and leads to considerable computational simplifications. It is possible that more sophisticated, potentially dependent, branch length priors would improve cluster inference overall. Priors based on models for epidemiological transmission trees, e.g. a birth-death model, might also be helpful. Given the often high recovery rates observed in the simulations, we are confident that the simplification was not overly detrimental. Improvements to the code should also make it possible to apply DM-PhyClus to much larger datasets, such as those collected for major HIV-1 genotyping programs. Right now, the main computational bottleneck is in the likelihood evaluations, with time complexity linear in the number of sites and sequences [30].

## Conclusion

We contend DM-PhyClus is a worthwhile addition to existing methods used to detect transmission clusters. Understanding clustering in epidemics is crucial: in the case of HIV-1 among men who have sex with men for example, transmission clusters have been found to contribute overwhelmingly to incidence [3, 6]. Investigations into the reasons behind the existence of those clusters are likely to help in reducing transmission rates, and those studies will need to rely on methods based on cluster definitions that reflect clinical insight, like DM-PhyClus.

## Appendix 1 - Algorithm description

The notation between brackets is used in Fig. 2.

**Input**:

1. **The data** (*y*_1_,…,*y*_*n*_): A sample of aligned DNA sequences,
2. **Topology** (*τ*, fixed): Can be, for example, the maximum likelihood topology,
3. **Nucleotide transition rate matrix** (*Q*, fixed): Can be an empirical estimate, like the one in [41], or alternatively, one derived from the sample itself, with the help of RAxML or MrBayes for example,
4. **Gamma shape parameter for among-sites mutation rate variation** (*r*, fixed): Assumed equal to the scale parameter, can be obtained in the same way as the nucleotide transition rate matrix. In the simulations, we use an estimate from [41],
5. **Number of among-sites rate variation categories** (*n*_*r*_, fixed).
6. **Cluster membership indices (*c***_**1**_**,…,*c***_***n***_**) prior**: Follows a Dirichlet-multinomial distribution, combined with a Poisson-distributed weight with a pre-determined rate parameter (*λ*, fixed), e.g. the number of clusters resulting from a conventional bootstrap-maximum likelihood phylogenetic clustering analysis,
7. **Poisson rate for the assumed number of clusters** (*λ*, fixed),
8. **Concentration parameter (*α*) prior**: Assumed gamma-distributed with user-specified shape (*η*, fixed) and scale parameters (*β*, fixed),
9. **Shape (*η*, fixed) and scale (*β*, fixed) parameter values for the concentration parameter prior**: We set the scale parameter equal to 0.1 in all analyses, and changed the shape parameter to vary the distributional mean,
10. **Transition kernel for the concentration parameter** (*α*): A uniform distribution with radius 0.5 centered at the current parameter value,
11. **Transition kernel for the cluster membership indices** (*c*_1_,…,*c*_*n*_): A uniform distribution over all configurations reachable from the current state. A configuration is reachable if it can be obtained by splitting in two a cluster of size 2 or more, or merging two neighbouring clusters. Two clusters are considered neighbours if their respective most recent common ancestors (MRCA) are siblings. Clusters are obtained by partitioning the sample into disjoint clades. It follows that each cluster can be represented, alternatively, by its MRCA. When a cluster is split in two, the MRCAs of the new clusters are the children nodes of the original cluster’s MRCA. When two neighbouring clusters are merged, the new cluster’s MRCA is the parent node of the selected two clusters’ MRCAs.
12. **Prior for branch lengths in the within-cluster phylogenies**$\left (l_{1}^{(w)}, \dots, l_{n(w)}^{(w)}\right)$: Assumed to follow the exponential distribution (*δ*),
13. **Prior for branch lengths in the between-cluster phylogeny**$\left (l_{1}^{(b)}, \dots, l_{k(k+1)/2}^{(w)}\right)$: Assumed to follow a log-normal distribution with equal mean (Mean) and standard deviation (SD, fixed), which implies a coefficient of variation of 1,
14. **Prior for the transition probabilities along branches in the within-cluster phylogenies**: Represented by an array of 4×4 matrices. Each row of the array corresponds to a different assumed mean branch length, while each column corresponds to a different rate variation category,
15. **Prior for the transition probabilities along branches in the between-cluster phylogeny**: Same as before,
16. **Starting value for the cluster membership indices** (*c*_1_,…,*c*_*n*_): Must be a partition of the sample into clades found in the input topology,
17. **Starting value for the Dirichlet-multinomial concentration parameter** (*α*),
18. **Starting values for the between-cluster and within-cluster transition probabilities**,
19. **Number of iterations**,
20. **Burn-in size**,
21. **Thinning ratio**.

**Algorithm output**:

1. Values sampled from the posterior distribution of the cluster membership indices (*c*_1_,…,*c*_*n*_),
2. Values sampled from the posterior distribution of the concentration parameter (*α*),
3. A non-standardized joint log-posterior probability value for the parameter values at the end of each iteration.

### A standard run

#### Obtaining the topology

In each simulation run, we start by obtaining an estimate of the maximum likelihood topology from RAxML. We assume that genetic distances follow the GTR+ *Γ*(5) model and use a subtype C outgroup (http://www.hiv.lanl.gov/, accession number: AB254141). We then produce 500 bootstrap estimates of the tree, resulting in the usual clade support estimates. RAxML stores the best scoring tree in a file with the “bestTree” mention. More details on RAxML’s tree optimization and scoring methods can be found in [48].

#### Starting values for the cluster membership indices

We then use the topology to obtain initial cluster estimates. More specifically, we look for a partition of the sample into clades for which,

1. Maximum patristic distance between any pair of elements within a clade is bounded above by an arbitrary value, e.g. 0.05 nt/bp,
2. Bootstrap support for any clade is above a certain value, e.g. 70%.

We find such a partition by traversing the tree starting at the root. At the beginning, all sequences are assumed to be in one cluster. If the (trivial) clade supported by the root node meets the requirements above, no further move is required. If not, we move down to the two children nodes, and update the cluster membership vector to account for the creation of a new cluster after the split of the original cluster into two non-overlapping clusters. At each child, we repeat the checks performed at the root, moving down and splitting clusters until a set that meets the clustering criteria is encountered, or until we reach a tip.

In the analyses, we impose a confidence requirement of 70%, and find cluster configurations for maximum genetic distance requirements between 0.03 nt/bp and 0.12 nt/bp. For each distance requirement, we have a potentially different set of clusters, and for each of them, we calculate the Dunn index [42], deriving the distance matrix from the phylogenetic estimate. Finally, we pick the set that maximizes that index as the starting value for the cluster membership indices.

#### Estimates of transition probabilities

Once we have an estimate of cluster membership indices, we use it to set up priors for transition probabilities along branches in the within-cluster and between-cluster phylogenies. In the within-cluster phylogenies, branch lengths have an exponential prior. We pick a range of values for the mean parameter by,

1. Computing the average branch length across all within-cluster phylogenies obtained from the starting partition,
2. Finding 20 equidistant points in a radius equal to 8% of the value computed previously.

For each point in the range, we simulate 100,000 values from the corresponding exponential distribution. We then obtain the required transition probability matrices by computing,

$$P^{(r)} = \sum\limits_{i=1}^{1e5} \exp({Qd}_{i} l_{r})/1e5, \qquad r = 1,2,3,$$

where *r* indexes the rate variation category, *d*_*i*_ denotes a value generated previously, *Q*, a transition rate matrix estimate, and *l*_*r*_, a distance scaling factor. We use a similar strategy to derive a prior distribution for transition probabilities along branches in the between-cluster phylogeny.

#### Running the chain and obtaining point estimates for cluster membership indices

Each iteration in the chain involves successive Metropolis-Hastings updates of the cluster membership indices, the between and within-cluster transition probabilities, and the concentration parameter. The algorithm produces a joint posterior probability value at the end of each iteration, which we use to identify the MAP estimate. To obtain the linkage-*xx* estimates, we compute an adjacency matrix from each sampled cluster membership vector, under the assumption that all sets of co-clustering sequences form fully-connected graphs, all disjoint from each other. We then average all adjacency matrices, and apply the *xx* threshold to the resulting matrix, rounding up to 1 all values in the matrix above the threshold, and down to 0 the other values. We then run the walktrap algorithm [40], using chains of 10 steps to detect disjoint sets, which correspond to the cluster membership indices estimate.

## Appendix 2 - Tuning parameters used in the simulations

### Simulating datasets

- Sample size: 200,
- Rate parameter for Poisson-distributed number of clusters: 50,
- Mean value for normally-distributed concentration parameter: 10,
- Standard deviation for normally-distributed concentration parameter: 2,
- Number of rate variation categories: 5,
- Shape and scale parameters for gamma-distributed rate variation: 0.7589,
- Number of datasets: 100,
- Root sequence: HXB2 sequence (http://www.hiv.lanl.gov/), sites 10-297 of the protease region (PR), and 112-741 of the reverse transcriptase (RT) region, of the *pol* gene.
- Limiting probabilities: (*A*=0.39,*T*=0.22,*C*=0.17,*G*=0.22)
- Rate matrix *Q*:
  $$\left[ \begin{array}{cccc} -0.83708096 & 0.04319486 & 0.12127074 & 0.67261536 \\ 0.07657272 & -0.82554421 & 0.66140131 & 0.08757018 \\ 0.27820934 & 0.85593111 & -1.18569748 & 0.05155703 \\ 1.19236359 & 0.08757018 & 0.03983952 & -1.31977330 \end{array} \right] $$
- Mean parameter for exponentially-distributed branch lengths in within-cluster phylogenies: 0.003,
- Mean and standard deviation parameters for log-normal-distributed branch lengths in between-cluster phylogenies: 0.008.

### Chain parameters

- Number of discrete states for the within-cluster and between-cluster transition probability matrices: 20,
- Number of samples used to obtain transition probability matrices: 100,000,
- Radius around mean within-cluster and between-cluster branch length estimates: 8%,
- Bootstrap confidence requirement for initial cluster estimate: 70%,
- Limiting probabilities: (*A*=0.4298969,*T*=0.2227602,*C*=0.1459,*G*=0.2014428),
- Rate matrix Q:
  $$\left[ \begin{array}{cccc} -0.79633415 & 0.04560603 & 0.10852696 & 0.64220116 \\ 0.08801344 & -0.76352160 & 0.59189771 & 0.08361045 \\ 0.31977658 & 0.90370975 & -1.27271206 & 0.04922573 \\ 1.37051455 & 0.09245841 & 0.03565297 & -1.49862593 \end{array} \right] $$
- Shape parameter for concentration parameter prior: 1000, 100, 10,
- Scale parameter for concentration parameter prior: 0.1,
- Poisson rate for weight applied to the cluster membership vector prior: 50,
- Number of iterations: 55,000.

## Appendix 3 - Tuning parameters used in the real data analysis

### Bootstrap analysis

- Number of discrete states for the within-cluster and between-cluster transition probability matrices: 20,
- Number of samples used to obtain transition probability matrices: 100,000,
- Radius around mean within-cluster and between-cluster branch length estimates: 8%,
- Discrete gamma distribution parameter: 0.7589,
- Bootstrap confidence requirement for initial cluster estimate: 70%,
- Limiting probabilities: (*A*=0.39,*T*=0.22,*C*=0.17,*G*=0.22),
- Rate matrix *Q*:
  $$\left[ \begin{array}{cccc} -0.83708096 & 0.04319486 & 0.12127074 & 0.67261536 \\ 0.07657272 & -0.82554421 & 0.66140131 & 0.08757018 \\ 0.27820934 & 0.85593111 & -1.18569748 & 0.05155703 \\ 1.19236359 & 0.08757018 & 0.03983952 & -1.31977330 \end{array} \right] $$
- Shape parameter for concentration parameter prior: 1000,
- Scale parameter for concentration parameter prior: 0.1,
- Poisson rate for weight applied to the cluster membership vector prior: 32,
- Number of iterations: 55,000.

### Approximation of the fully Bayesian analysis

- Number of discrete states for the within-cluster and between-cluster transition probability matrices: 20,
- Number of samples used to obtain transition probability matrices: 100,000,
- Radius around mean within-cluster and between-cluster branch length estimates: 8%,
- Discrete gamma distribution parameter: 0.4394492,
- Limiting probabilities: (*A*=0.4032267,*T*=0.2147781,*C*=0.1625374,*G*=0.2194578),
- Rate matrix *Q*:
  $$\left[ \begin{array}{cccc} -0.8411512 & 0.05921394 & 0.11223579 & 0.66970147 \\ 0.1111689 & -0.80528701 & 0.62140549 & 0.07271263 \\ 0.2784372 & 0.82112972 & -1.17182113 & 0.07225417 \\ 1.2304940 & 0.07116212 & 0.05351373 & -1.35516988 \end{array} \right] $$
- Shape parameter for concentration parameter prior: 1000,
- Scale parameter for concentration parameter prior: 0.1,
- Poisson rate for weight applied to the cluster membership vector prior: 32,
- Number of iterations: 55,000.

### Main run

- Number of discrete states for the within-cluster and between-cluster transition probability matrices: 20,
- Number of samples used to obtain transition probability matrices: 100,000,
- Radius around mean within-cluster and between-cluster branch length estimates: 8%,
- Discrete gamma distribution parameter: 0.7589,
- Bootstrap confidence requirement for initial cluster estimate: 70%,
- Limiting probabilities: (*A*=0.39,*T*=0.22,*C*=0.17,*G*=0.22),
- Rate matrix *Q*:
  $$\left[ \begin{array}{cccc} -0.83708096 & 0.04319486 & 0.12127074 & 0.67261536 \\ 0.07657272 & -0.82554421 & 0.66140131 & 0.08757018 \\ 0.27820934 & 0.85593111 & -1.18569748 & 0.05155703 \\ 1.19236359 & 0.08757018 & 0.03983952 & -1.31977330 \end{array} \right] $$
- Shape parameter for concentration parameter prior: 1000,
- Scale parameter for concentration parameter prior: 0.1,
- Poisson rate for weight applied to the cluster membership vector prior: 32,
- Number of iterations: 220,000.

## Appendix 4 - Notes on the software

We implemented DM-PhyClus mostly in R, with C++ modules to handle log-likelihood evaluations. In R, we use classes and functions defined in the *ape* and *phangorn* packages [43] to represent and manipulate phylogenies. The interface between R and C++ relies on features offered by the *Rcpp* and *RcppArmadillo* packages [45, 46].

Unsurprisingly, the C++ modules make extensive use of containers in the Standard Template Library (STL) and functionalities implemented in the C++11 standard. For now, the code still relies on the GNU Scientific Library (GSL) for random number generation, but we intend to change that in future versions in order to improve portability. Phylogenies are represented by a custom binary tree class, consisting of objects instanced from an input node class, representing the tips of the tree, and from an internal node class. Both classes inherit from an abstract class, standing in for a generic tree node.

We use Felsenstein’s tree-pruning algorithm [38] to perform likelihood evaluations. Our implementation of the latter algorithm makes use of containers, functions, and operators defined in the Armadillo library [49]. To reduce the algorithm’s memory footprint and improve performance, all intermediate solutions are saved in a map container, and the tree node objects store merely a pointer to the corresponding map elements. To ensure pointer validity, we opted for an ordered map. We use functions in the *boost* package in the generation of keys for map elements. The keys are obtained recursively by combining, among other things, keys computed for children nodes.

The size of the map tends to increase quickly for even moderately-sized datasets, eventually saturating the memory on most standard machines, and so, the software wipes the map periodically. That strategy is also beneficial from a computational standpoint: by eliminating configurations rarely visited by the algorithm, mean lookup time is reduced. Moreover, allowing very large maps is detrimental from a computational standpoint: once a map reaches a certain size, re-computing solutions turns out to be on average faster than doing a lookup.

We obtained a great boost in performance after defining a persistent pointer to the object used to represent the tree structure. Indeed, profiling had revealed that the software was being weighed down considerably by the memory allocation operations involved in building the tree structure, hence the vast improvement resulting from keeping the object in memory and updating it when required. More specifically, we implemented that strategy by passing a so-called *external pointer* to R, implemented by the XPtr class template in the Rcpp library. By trading the pointer between R and C++, we effectively prevent garbage collection of the tree object until the pointer goes out of scope.

We wrote a vignette that explains how the R package can be used to cluster an arbitrary dataset.

## Appendix 5 - Log-posterior probability graph

See Fig. 4.

**Fig. 4:**
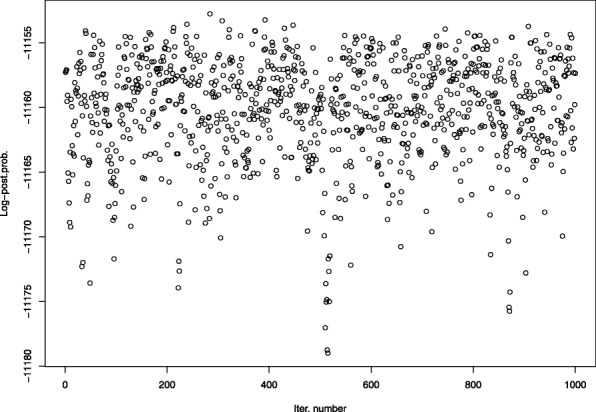
Log-posterior probability graph for the thinned chain obtained from one of the simulated samples

## Additional files

Additional file 1 This file contains an R object giving the results for the scenario in the simulation study where the concentration parameter prior is assumed to have mean 100 and we use the MAP topology. The file is compressed in gzip format. (GZ 8234 kb)

Additional file 2 This file contains an R object giving the results for the scenario in the simulation study where the concentration parameter prior is assumed to have mean 100 and we use the ML topology. The file is compressed in gzip format. (GZ 7658 kb)

Additional file 3 This file contains an R object giving the results for the scenario in the simulation study where the concentration parameter prior is assumed to have mean 10 and we use the MAP topology. The file is compressed in gzip format. (GZ 8365 kb)

Additional file 4 This file contains an R object giving the results for the scenario in the simulation study where the concentration parameter prior is assumed to have mean 10 and we use the ML topology. The file is compressed in gzip format. (GZ 8151 kb)

Additional file 5 This file contains an R object giving the results for the scenario in the simulation study where the concentration parameter prior is assumed to have mean 1 and we use the MAP topology. The file is compressed in gzip format. (GZ 8412 kb)

Additional file 6 This file contains an R object giving the results for the scenario in the simulation study where the concentration parameter prior is assumed to have mean 1 and we use the ML topology. The file is compressed in gzip format. (GZ 8293 kb)

## References

[1] Foley BT, Leitner TK, Korber BTM, Apetrei C, Hahn B, Mizrachi I, Mullins J, Rambaut A, Wolinsky S. HIV sequence compendium 2013;2013.

[2] Huerta-Cepas J, Capella-Gutiérrez S, Pryszcz LP, Marcet-Houben M, Gabaldón T (2014). PhylomeDB v4: zooming into the plurality of evolutionary histories of a genome. Nucleic Acids Res.

[3] Brenner BG, Wainberg MA (2013). Future of phylogeny in HIV prevention. J Acquir Immune Defic Syndr.

[4] Brenner B, Wainberg MA, Roger M (2013). Phylogenetic inferences on HIV-1 transmission: implications for the design of prevention and treatment interventions. AIDS.

[5] Van der Spoel van Dijk A, Makhoahle PM, Rigouts L, Baba K (2016). Diverse molecular genotypes of Mycobacterium tuberculosis complex isolates circulating in the Free State, South Africa. Int J Microbiol.

[6] Brenner BG, Roger M, Stephens DA, Moisi D, Hardy I, Weinberg J, Turgel R, Charest H, Koopman J, Wainberg MA, Montreal PHI Cohort Study Group (2011). Transmission clustering drives the onward spread of the HIV epidemic among men who have sex with men in Quebec. J Infect Dis.

[7] Brenner BG, Roger M, Routy J-P, Moisi D, Ntemgwa M, Matte C, Baril J-G, Thomas R, Rouleau D, Bruneau J, Leblanc R, Legault M, Tremblay C, Charest H, Wainberg MA, Quebec Primary HIV Infection Study Group (2007). High rates of forward transmission events after acute/early HIV-1 infection. J Infect Dis.

[8] Chalmet K, Staelens D, Blot S, Dinakis S, Pelgrom J, Plum J, Vogelaers D, Vandekerckhove L, Verhofstede C (2010). Epidemiological study of phylogenetic transmission clusters in a local HIV-1 epidemic reveals distinct differences between subtype B and non-B infections. BMC Infect Dis.

[9] Villandre L, Stephens DA, Labbe A, Günthard HF, Kouyos R, Stadler T, Swiss HIV Cohort Study (2016). Assessment of overlap of phylogenetic transmission clusters and communities in simple sexual contact networks: Applications to HIV-1. PLoS One.

[10] Yang Z, Rannala B (1997). Bayesian phylogenetic inference using DNA sequences: a Markov Chain Monte Carlo method. Mol Biol Evol.

[11] Ronquist F, Teslenko M, van der Mark P, Ayres DL, Darling A, Höhna S, Larget B, Liu L, Suchard MA, Huelsenbeck JP (2012). MrBayes 3.2: Efficient Bayesian phylogenetic inference and model choice across a large model space. Syst Biol.

[12] Drummond AJ, Suchard MA, Xie D, Rambaut A (2012). Bayesian phylogenetics with BEAUti and the BEAST 1.7. Mol Biol Evol.

[13] Bouchard-Côté A, Sankararaman S, Jordan MI (2012). Phylogenetic inference via sequential Monte Carlo. Syst Biol.

[14] Cheon S, Liang F (2008). Phylogenetic tree construction using sequential stochastic approximation Monte Carlo. Biosystems.

[15] Tamura K, Stecher G, Peterson D, Filipski A, Kumar S (2013). MEGA6: Molecular evolutionary genetics analysis version 6.0. Mol Biol Evol.

[16] Swofford DL. PAUP*: Phylogenetic Analysis Using Parsimony (and Other Methods). Sinauer Associates. 2001. http://paup.sc.fsu.edu/about.html. Accessed 21 June 2017.

[17] Stamatakis Alexandros (2014). RAxML version 8: a tool for phylogenetic analysis and post-analysis of large phylogenies. Bioinformatics.

[18] Price MN, Dehal PS, Arkin AP (2010). FastTree 2 – approximately maximum-likelihood trees for large alignments. PLoS ONE.

[19] Erixon P, Svennblad B, Britton T, Oxelman B (2003). Reliability of Bayesian posterior probabilities and bootstrap frequencies in phylogenetics. Syst Biol.

[20] Susko E (2009). Bootstrap support is not first-order correct. Syst Biol.

[21] Makarenkov V, Boc A, Xie J, Peres-Neto P, Lapointe F-J, Legendre P (2010). Weighted bootstrapping: a correction method for assessing the robustness of phylogenetic trees. BMC Evol Biol.

[22] Larget B, Simon DL (1999). Markov chain Monte Carlo algorithms for the Bayesian analysis of phylogenetic trees. Mol Biol Evol.

[23] Bryant D (2003). A classification of consensus methods for phylogenetics. DIMACS Ser Discret Math Theor Comput Sci.

[24] Holder MT, Sukumaran J, Lewis PO (2008). A justification for reporting the majority-rule consensus tree in Bayesian phylogenetics. Syst Biol.

[25] Prosperi MCF, Ciccozzi M, Fanti I, Saladini F, Pecorari M, Borghi V, Giambenedetto SD, Bruzzone B, Capetti A, Vivarelli A, Rusconi S, Re MC, Gismondo MR, Sighinolfi L, Gray RR, Salemi M, Zazzi M, Luca AD, on behalf of the ARCA collaborative group (2011). A novel methodology for large-scale phylogeny partition. Nat Commun.

[26] Ragonnet-Cronin M, Hodcroft E, Hué S, Fearnhill E, Delpech V, Brown AJL, Lycett S (2013). Automated analysis of phylogenetic clusters. BMC Bioinforma.

[27] Jukes TH, Cantor CR (1969). Evolution of protein molecules. Mamm Protein Metab.

[28] Kimura M (1980). A simple method for estimating evolutionary rates of base substitutions through comparative studies of nucleotide sequences. J Mol Evol.

[29] Hasegawa M, Kishino H, Yano T (1985). Dating of the human-ape splitting by a molecular clock of mitochondrial DNA. J Mol Evol.

[30] Yang Z (2006). Computational Molecular Evolution. Oxford Series in Ecology and Evolution.

[31] Ahumada-Ruiz S, Flores-Figueroa D, Toala-González I, Thomson MM (2009). Analysis of HIV-1 pol sequences from Panama: identification of phylogenetic clusters within subtype B and detection of antiretroviral drug resistance mutations. Infect Genet Evol.

[32] Chaix M-L, Descamps D, Harzic M, Schneider V, Deveau C, Tamalet C, Pellegrin I, Izopet J, Ruffault A, Masquelier B, Meyer L, Rouzioux C, Brun-Vezinet F, Costagliola D (2003). Stable prevalence of genotypic drug resistance mutations but increase in non-B virus among patients with primary HIV-1 infection in France. AIDS.

[33] Ibe S, Hattori J, Fujisaki S, Shigemi U, Fujisaki S, Shimizu K, Nakamura K, Kazumi T, Yokomaku Y, Mamiya N, Hamaguchi M, Kaneda T (2008). Trend of drug-resistant HIV type 1 emergence among therapy-naive patients in Nagoya, Japan: an 8-year surveillance from 1999 to 2006. AIDS Res Hum Retrovir.

[34] Lindström A, Ohlis A, Huigen M, Nijhuis M, Berglund T, Bratt G, Sandström E, Albert J (2006). HIV-1 transmission cluster with M41L ’singleton’ mutation and decreased transmission of resistance in newly diagnosed Swedish homosexual men. Antivir Ther.

[35] Leigh Brown AJ, Lycett SJ, Weinert L, Hughes GJ, Fearnhill E, Dunn DT, the UK HIV Drug Resistance Collaboration (2011). Transmission network parameters estimated from HIV sequences for a nationwide epidemic. J Infect Dis.

[36] Vrbik I, Stephens DA, Roger M, Brenner BG (2015). The Gap procedure: for the identification of phylogenetic clusters in HIV-1 sequence data. BMC Bioinforma.

[37] Hastings WK (1970). Monte Carlo sampling methods using Markov chains and their applications. Biometrika.

[38] Felsenstein J (1981). Evolutionary trees from DNA sequences: A maximum likelihood approach. J Mol Evol.

[39] Kouyos RD, von Wyl V, Yerly S, Böni J, Rieder P, Joos B, Taffé P, Shah C, Bürgisser P, Klimkait T, Weber R, Hirschel B, Cavassini M, Rauch A, Battegay M, Vernazza PL, Bernasconi E, Ledergerber B, Bonhoeffer S, Günthard HF, Swiss HIV Cohort Study (2011). Ambiguous nucleotide calls from population-based sequencing of HIV-1 are a marker for viral diversity and the age of infection. Clin Infect Dis.

[40] Pons P, Latapy M. Computing communities in large networks using random walks. ArXiv Phys e-prints. 2005. http://arxiv.org/abs/physics/0512106physics/0512106. Accessed 30 Aug 2016.

[41] Posada D, Crandall KA (2001). Selecting models of nucleotide substitution: an application to human immunodeficiency virus 1 (HIV-1). Mol Biol Evol.

[42] Dunn JC (1973). A fuzzy relative of the ISODATA Process and Its Use in Detecting Compact Well-Separated Clusters. Cybern Syst.

[43] Schliep KP (2011). phangorn: phylogenetic analysis in R. Bioinformatics.

[44] Revell LJ (2012). phytools: An R package for phylogenetic comparative biology (and other things). Methods Ecol Evol.

[45] Eddelbuettel D, François R (2011). Rcpp: Seamless R and C++ integration. J Stat Softw.

[46] Eddelbuettel D (2013). Seamless R and C++ Integration With Rcpp.

[47] Chen M-H, Kuo L, Lewis PO. Bayesian Phylogenetics: methods, algorithms, and applications.CRC Press; 2014, pp. 5–23.

[48] Stamatakis A, Ludwig T, Meier H (2005). RAxML-III: a fast program for maximum likelihood-based inference of large phylogenetic trees. Bioinformatics.

[49] Sanderson C, Curtin R (2016). Armadillo: a template-based C++ library for linear algebra. J Open Source Softw.

